# Early Pain Relief in Malignant Psoas Syndrome with Radiation Therapy: A Case Report

**DOI:** 10.7759/cureus.73007

**Published:** 2024-11-04

**Authors:** Masafumi Uno, Yukihisa Tamaki, Hiroshi Burioka, Natsuko Nagano, Yoko Sonoyama

**Affiliations:** 1 Radiation Oncology, Shimane University Faculty of Medicine, Izumo, JPN

**Keywords:** lumbosacral plexopathy, malignant psoas syndrome, mps, pain management, palliative care, palliative radiotherapy, radiation therapy

## Abstract

Malignant psoas syndrome is caused by a malignant tumor infiltrating the psoas muscle and is characterized by severe pain. Currently, no definitive diagnostic or therapeutic approaches have been established for this condition. Although multiple medications are often used for pain relief, pain management can often be challenging, and there are various treatment options. Here, we report a case of a Japanese man in his 60s who was diagnosed with malignant psoas syndrome due to metastasis of myxoid liposarcoma. Despite undergoing several pharmacological treatments for severe pain, their effects were insufficient. Palliative radiation therapy was therefore planned and started to relieve pain. We discussed radiation therapy methods. Since there was no previous literature on treatment with single doses exceeding 3 Gy and the attending physician expected the patient to have a little longer survival time, we decided to treat 39 Gy in 13 fractions of radiation therapy. Early pain relief was achieved with 24 Gy in eight fractions. Radiation therapy was continued without change after the pain improved. The treatment was terminated at 36 Gy in 12 fractions due to the deterioration of the patient’s general condition caused by the progression of metastases throughout the body. The patient died two days after the end of his treatment (18 days after the start of radiation therapy) due to exacerbation of his primary disease. No adverse events related to radiation therapy were observed. In this case, radiation therapy was found to be effective at an early stage in relieving pain from malignant psoas syndrome, which was difficult to control with multiple pharmacological treatments. Given its effectiveness in early pain relief without adverse events, radiation therapy should be actively considered as a treatment option for malignant psoas syndrome.

## Introduction

Ipsilateral lumbosacral plexopathy caused by malignant tumor infiltration of the psoas muscle is referred to as malignant psoas syndrome (MPS). This syndrome was first reported by Stevens and Gonet in 1990 [1]. In their report, they identified three key characteristics of MPS: (i) symptoms indicating proximal (L1-L4) lumbar plexopathy, (ii) painful flexion of the ipsilateral hip, and (iii) computed tomography (CT) or pathologically evident malignant infiltration of the psoas major muscle.

The pain associated with MPS is thought to result from a combination of nociceptive pain and neuropathic pain, which makes the syndrome particularly painful. Due to this complex nature, pain management is often challenging, requiring a variety of treatments [2]. However, there is still no clear consensus on the clinical symptoms, diagnostic criteria, or therapeutic approach for MPS. Pharmacological treatment is the most common option for managing the pain in MPS. However, even with the use of multiple medications, satisfactory pain relief is often not achieved [2,3]. Other treatment options that have been reported include radiation therapy [1,2,4-8], surgery [9], chemotherapy [4], and nerve block [6].

Since there is no established treatment protocol for MPS, physicians must choose the most effective treatment based on the individual patient's condition. Here, we report a case of MPS in which pain was difficult to control with multiple medications but was alleviated early following radiation therapy.

## Case presentation

The patient was a 69-year-old Japanese man. Four years earlier, he was diagnosed with primary myxoid liposarcoma in the right popliteal fossa. The initial clinical stage was cT4N0M0, cStage IB (Union for International Cancer Control (UICC), 8th edition, 2016) and he was treated with surgery. The grade of malignancy was low grade. The pathologic stage of this patient was pT4N0M0 pStageIB (UICC 2017). Intraoperative rapid diagnosis was positive for transection, but additional resection was performed and the margins were negative. However, because there was a non-contiguous tumor in the extracapsular fatty tissue, the pathologist evaluated it as requiring careful follow-up. Postoperative radiation therapy was performed (50 Gy in 25 fractions) due to the inability to perform a sufficiently extensive resection and the pathology results. Since then, the disease recurred multiple times at sites including the lung and liver. The recurrences were treated with surgery and radiation therapy. Five months prior to the current presentation, distant metastases were found throughout his body, but the patient chose not to undergo chemotherapy, so his treatment plan was shifted to the best supportive care.

The patient presented with a recent complaint of severe pain in the anterior part of the left thigh. His pain was mild when he was at rest, but very severe when he moved. Due to the pain, he was unable to move his left thigh and exhibited fixed flexion of the left hip. The numeric rating scale (NRS) score for his dynamic pain was 6/10. CT revealed numerous metastatic lesions throughout the body, including multiple peritoneal dissemination in the abdominal and pelvic regions, multiple liver metastases, and pleural effusion caused by thoracic metastasis. Additionally, one of the peritoneal metastases had infiltrated the left psoas muscle. Through consultation between the medical oncologist, orthopedic surgeon, and palliative care physician, the patient was clinically diagnosed with MPS. At this point, the patient's condition met all three characteristics of MPS reported by Stevens and Gonet [1]. The patient’s pain was severe, and he had been using multiple analgesics. While the medication improved his rest pain, it was insufficient to control his dynamic pain. A consultation was therefore made with the radiation oncology department to assess the suitability of radiation therapy for pain relief.

**Figure 1:**
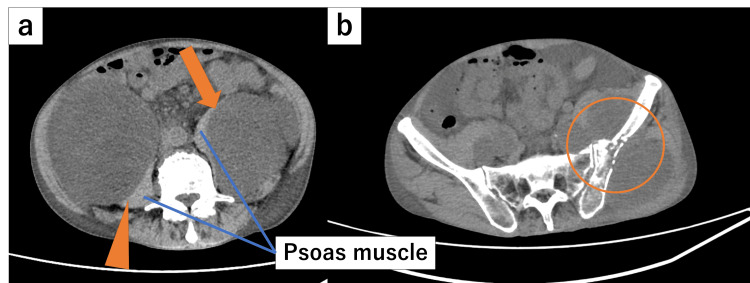
Chest and abdominal computed tomography in the axial plane before radiation therapy. (a) A large disseminated lesion infiltrating the left psoas muscle was identified as the cause of malignant psoas syndrome (arrow). Another disseminated lesion infiltrated the right psoas muscle (arrowhead), but there were no symptoms on the right side. (b) A separate disseminated lesion infiltrating the left iliac bone. The patient experienced left buttock pain (circle).

We determined that this pain was associated with MPS and was caused by the peritoneal metastasis infiltrating the left psoas muscle (Figure 1a). The patient also had left buttock pain, which was thought to be attributable to a separate peritoneal metastasis infiltrating the left iliac bone (Figure 1b). It was determined that radiation therapy was appropriate for pain relief for both of these lesions. Although another peritoneal metastasis had infiltrated the right psoas muscle, there was no pain on the right side, so no radiation therapy was administered to that area.

**Figure 2:**
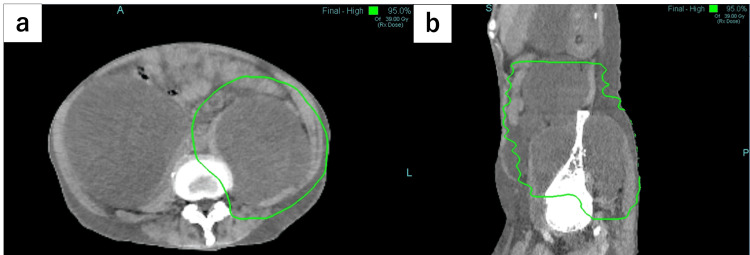
Dose distribution of radiation therapy. The green line indicates the 95% isodose line. (a) Dose distribution on an axial image. (b) Dose distribution on a sagittal image.

To alleviate the two areas of pain on the left side, we decided to include both metastatic lesions infiltrating the left psoas muscle and the one infiltrating the left iliac bone within the radiation field. As a result, the radiation field extended approximately 28 cm craniocaudally. The optimal radiation therapy regimen for MPS has not been established. Since there had been no previous literature on treatment with single doses exceeding 3 Gy and the attending physician expected the patient to have a longer survival time, we discussed with the attending physician and decided to treat the patient with 39 Gy in 13 fractions. The biological equivalent dose (α/β = 10) of radiation therapy was 50.7 Gy. The dose distribution is shown in Figure 2. The patient had received three previous radiation treatments: the first in the right popliteal fossa (50 Gy in 25 fractions), the second in the chest (66 Gy in 33 fractions), and the third in the cervical spine (39 Gy in 13 fractions). However, none of the prior radiation fields were close to the current one, so there were no issues with proceeding with this course of treatment. Radiation therapy was performed daily.

At the time of his visit to the radiation oncology department, he had an Eastern Cooperative Oncology Group (ECOG) performance status of 4 and was transported via stretcher. He was receiving multiple analgesics intravenously, including 60 mg/day of oxycodone hydrochloride hydrate, 3000 mg/day of acetaminophen, and 100 mg/day of flurbiprofen axetil. Although his rest pain improved with medication, he continued to experience severe dynamic pain in his left hip. The NRS score for his rest pain was 4/10, whereas the NRS score for his dynamic pain was 6/10.

Due to the pain, he presented with fixed flexion of his left hip, a characteristic finding of MPS. The patient was willing to move, but the pain on movement made it impossible. Other symptoms related to the progression of the metastatic lesions throughout his body were also observed. He experienced dyspnea due to pleural dissemination and pleural effusion and was receiving 2 L/minute of oxygen. While the oxygen relieved his dyspnea at rest, he still experienced shortness of breath on movement.

He also exhibited abdominal distention due to multiple liver metastases and peritoneal dissemination, along with hypoalbuminemia and edema in both legs. Furthermore, he complained of fatigue. The patient's position during radiation therapy was such that both legs were slightly elevated and both hips were fixed in a flexed position to reduce pain during radiation therapy. Although the patient’s general condition was poor, radiation therapy was promptly initiated and continued without any problems. After receiving 24 Gy, his pain improved, and he stated that the radiation therapy had been very beneficial. His NRS score for dynamic pain before radiation therapy was 6-7/10, which decreased to 1-2/10 at the time he had completed 24 Gy. In addition, flexion fixation of the left hip was released and he did not complain of pain during hip extension. The radiation therapy was continued without change after the pain improved.

However, systemic metastases gradually progressed. His hypoalbuminemia and bilateral leg edema worsened, and exudate began to appear in his right leg. His oxygen requirements also increased. By the end of 36 Gy, he began to show signs of lethargy due to the deterioration of his overall condition, leading to the termination of radiation therapy. The patient’s condition continued to decline after the conclusion of radiation therapy, and he died two days after ending treatment (18 days after the start of radiation therapy) due to exacerbation of his primary disease. There were no adverse events related to the radiation therapy. Radiation therapy resulted in dramatic pain relief from the severe pain caused by MPS.

## Discussion

MPS is a painful condition caused by malignant tumor infiltration of the psoas muscle [1]. The first report on MPS outlined three characteristics: (i) symptoms indicating proximal (L1-L4) lumbar plexopathy, (ii) painful flexion of the ipsilateral hip, and (iii) CT or pathologically evident malignant infiltration of the psoas major muscle [1]. In our case, the patient exhibited anterior left thigh pain, fixed flexion of the left hip, and CT findings of psoas muscle infiltration, which led to a clinical diagnosis of MPS after multidisciplinary consultation between the medical oncologist, orthopedic surgeon, and palliative care physician. These symptoms met the three characteristics described in the original report. However, Agar et al. found that only 13 out of 23 reported cases met all three criteria [2]. Another recent review article also pointed out that not all MPS cases present with fixed flexion of the hip [10]. Thus, there are currently no definitive diagnostic criteria for MPS. This syndrome is considered extremely rare [10]. Even when non-MPS cases are included, the incidence of lumbosacral plexopathy in cancer patients is reported to be as low as 0.71% [11]. However, Takamatsu et al. reported that they encountered three cases of MPS associated with gynecological tumors in their hospital within a year [4]. They suggested that MPS might be underdiagnosed due to the lack of general recognition, implying that more cases likely exist. Proper diagnosis is crucial to ensure that patients receive appropriate treatment; thus, it is necessary to establish diagnostic criteria and actively share case information.

Pain is generally caused by either nociceptive pain or neuropathic pain. However, pain in MPS is reported to arise from a combination of nociceptive pain due to tumor infiltration into the psoas muscle, causing muscle spasms, and neuropathic pain from lumbosacral plexopathy [1,3,7]. This dual pain mechanism results in intense pain, a hallmark of MPS. Pain management is crucial in palliative care, as pain negatively impacts cancer patients' quality of life (QoL) and is linked to depression [12]. In our case, the patient’s rest pain improved with pharmacological intervention, but dynamic pain persisted, significantly lowering his QoL due to impaired mobility.

Since MPS pain involves both nociceptive and neuropathic components, multimodal treatments are often required [1,3,7]. Pharmacological treatments, including opioid and non-opioid analgesics as well as analgesic adjuvants (e.g., neuropathic agents or muscle relaxants), are typically first-line therapies. However, achieving adequate pain control is often challenging due to limited effectiveness [2,3]. In our case, despite the use of multiple medications, including oxycodone hydrochloride hydrate, acetaminophen, and flurbiprofen axetil, the rest pain improved but dynamic pain remained insufficiently controlled, necessitating radiation therapy as the next treatment option.

There are several reports of radiation therapy for MPS [1,2,4-8]. The dose fractions of radiation therapy in these case reports and our case is listed (Table 1). Radiation therapy has been reported as effective for MPS-related pain. Suraj et al. reviewed 13 cases where radiation therapy provided pain relief, six of which had no response to pharmacological treatment alone [10]. Similarly, in our case, radiation therapy was effective in alleviating pain, and pain relief was achieved. Although there have been several reports of radiation therapy being effective, the optimal method of radiation therapy (dose fractionations, target, etc.) has not yet been established.

**Table 1:** List of MPS patients who received radiation therapy. Radiation therapy for patients 1, 11, and 21 was terminated midway through the course of the treatment. Patient number 7 was treated first on the left side and second on the right side. M: male; F: female; RT: radiation therapy; MPS: malignant psoas syndrome

Patient Number	Reference	Age (year) and Sex	Primary Cancer	Dose (Gy)	Number of fractions	Total dose (Gy)
1	[1]	15 M	Bladder cancer	1.5	2	3
2	[1]	60 F	Bladder cancer	None Stated	None Stated	50
3	[1]	81 F	Prostate cancer	None Stated	None Stated	50
4	[2]	49 F	Non-Hodgkin lymphoma	None Stated	None Stated	None Stated
5	[4]	31 F	Cervical cancer	1.8	25	45
6	[4]	63 F	Uterine serous carcinoma	2.5	16	40
7a (the first RT)	[5]	49 F	Ovarian cancer	3	13	39
7b (the second RT)	[5]	49 F	Ovarian cancer	3	13	39
8	[5]	74 F	Endometrial cancer	1.8	30	54
9	[5]	75 F	Cervical cancer	1.8	33	59.4
10	[5]	47 M	Urachal cancer	2	30	60
11	[5]	54 F	Cervical cancer	3	7	21
12	[5]	44 F	Cervical cancer	1.8	33	59.4
13	[5]	61 M	Ureteral cancer	3	10	30
14	[5]	71 M	Rectal cancer	1.8	30	54
15	[5]	70 M	Bladder cancer	3	10	30
16	[5]	73 F	Gastric cancer	3	13	39
17	[5]	80 M	Bladder cancer	3	10	30
18	[6]	45 F	Gastric cancer	3	10	30
19	[7]	68 F	Low-grade sarcoma	1.8	28	50.4
20	[8]	35 F	Urachal cancer	2.5	15	37.5
21	Our case	69 M	Myxoid liposarcoma	3	12	36

Our decision for this radiation therapy regimen was based on two main reasons. The first was a search of the past literature [1,2,4-8], we could not find any treatments with single doses exceeding 3 Gy. Second, prior to the start of radiation therapy, the attending physician had expected him to live a little longer. Considering these factors, we discussed with the attending physician and decided to treat the patient with 39 Gy in 13 fractions. However, it is possible that this treatment was not the best approach for this patient, given the fact that pain relief was obtained early in the treatment and that 13 treatments could not be completed. Given that pain relief was obtained before the end of irradiation, we think that radiation therapy with a shorter treatment period is worth trying. Finding better methods of radiation therapy for MPS is a challenge for the future.

Reports on the effects of the timing of radiation therapy in MPS are limited, but Sanuki et al. reported that all 11 cases of MPS studied experienced pain relief by the end of radiation therapy [5]. This aligns with our case, where early pain relief was observed. In addition, the study by Sanuki et al. included tumors that do not have high radiosensitivity, such as bladder cancer and endometrial cancer, but they were able to obtain early and effective pain relief as with other histological types [5]. Therefore, it is thought that a mechanism other than tumor shrinkage contributes to pain relief, but the clear mechanism by which radiation therapy alleviates MPS pain remains unclear.

It has been reported that when radiation therapy is applied to bone metastases, pain can improve within 24 hours, suggesting that a very rapid response may be related to the presence of highly radiosensitive inflammatory cells [13]. Shindo et al. also reported a similar response to radiation therapy aimed at pain relief for non-bone lesions, suggesting a potential common mechanism between bone and non-bone lesions, which may also apply to MPS [14]. The prognosis of MPS is generally poor [4]. In our case as well, the patient died 18 days after the initiation of radiation therapy. Since radiation therapy can provide early pain relief, it should be actively considered as a pain management option for MPS, which generally has a poor prognosis and is challenging in terms of pain control. 

## Conclusions

We encountered a case of MPS in which early pain relief was achieved through radiation therapy, despite difficulty controlling the pain with medication. Radiation therapy had no adverse effects and provided early, effective pain relief. Therefore, radiation therapy should be actively considered as a treatment option for managing MPS.
